# In vitro investigation of the cleaning efficacy, shaping ability, preparation time and file deformation of continuous rotary, reciprocating rotary and manual instrumentations in primary molars

**DOI:** 10.15171/joddd.2016.008

**Published:** 2016-03-16

**Authors:** Nahid Ramazani, Abbas Mohammadi, Foroogh Amirabadi, Mohsen Ramazani, Farzane Ehsani

**Affiliations:** ^1^Associate Professor, Children and Adolescents Health Research Center, Oral and Dental Diseases Research Center, Department of Pediatric Dentistry, Dental School, Zahedan University of Medical Sciences, Zahedan, Iran; ^2^Assistant Professor, Oral and Dental Diseases Research Center, Department of Oral and Maxillofacial Radiology, Dental School, Zahedan University of Medical Sciences, Zahedan, Iran; ^3^Assistant Professor, Children and Adolescents Health Research Center, Oral and Dental Diseases Research Center, Department of Pediatric Dentistry, Dental School, Zahedan University of Medical Sciences, Zahedan, Iran; ^4^Assistant Professor, Department of Endodontics, Dental School, Mazandaran University of Medical Sciences, Sari, Iran; ^5^Dentist, Zahedan University of Medical Sciences, Zahedan, Iran

**Keywords:** Deciduous teeth, pulpectomy, root canal preparation

## Abstract

***Background. ***Efficient canal preparation is the key to successful root canal treatment. This study aimed to assess the cleaning and shaping ability, preparation time and file deformation of rotary, reciprocating and manual instrumentation in canal preparation of primary molars.

***Methods.*** The mesiobuccal canals of 64 extracted primary mandibular second molars were injected with India ink. The samples were randomly divided into one control and three experimental groups. Experimental groups were instrumented with K-file, Mtwo in continuous rotation and Reciproc in reciprocating motion, respectively. The control group received no treatment. The files were discarded after four applications. Shaping ability was evaluated using CBCT. After clearing, ink removal was scored. Preparation time and file fracture or deformation was also recorded. Data were analyzed with SPSS 19 using chi-squared, Fisher’s exact test, Kruskal-Wallis and post hoc tests at a significance level of 0.05.

***Results.*** Considering cleanliness, at coronal third Reciproc was better than K-file (P < 0.001), but not more effective than Mtwo (P = 0.080). Furthermore, Mtwo leaved the canal cleaner than K-file (P = 0.001). In the middle third, only Reciproc exhibited better cleaning efficacy than K-file (P = 0.005). In the apical third, no difference was detected between the groups (P = 0.794). Regarding shaping ability, no differences were found between Reciproc and Mtwo (P = 1.00). Meanwhile, both displayed better shaping efficacy than K-file (P < 0.05). Between each two groups, there were differences in preparation time (P < 0.05), with Reciproc being the fastest. No file failure occurred.

***Conclusion.*** Fast and sufficient cleaning and shaping could be achieved with Mtwo and especially with Reciproc.

## Introduction

Pediatric endodontics aims to maintain the integrity and health of the oral tissues.^1-3^ One treatment in this context is pulpectomy. In addition to removal of the inflamed or necrotic pulp, the term pulpectomy in pediatric dentistry implies cleaning and shaping the root canal as well.^4^ Elimination of organic debris through instrumentation is one of the most critical parts in successful pulpectomy procedure.^5-8^ Considering this fact, pulpectomy of posterior teeth is a challenge for clinicians who treat children.^1,9^ Difficulties in instrumentation of canals with complexities, lengthy endodontic preparation and some behavior problems are challenging and require advances to update techniques.^2,9,10^

Methods of mechanical preparation include manual preparation, sonic and ultrasonic instrumentation, and automated systems.^2,5,7^ The use of engine-driven NiTi instruments has been introduced to dentistry with increasing frequency.^11,12^ These instruments were originally designed for use with continuous rotation movements.^13^ These instruments promote adequate canal shapes^14,15^ and nowadays are commonly used in endodontic treatment.^16^ Barr et al introduced rotary instrumentation of primary teeth.^7,10^

Rotary NiTi instruments create more rapid preparation and more centered smooth predetermined canal shapes, promoting a more uniform filling, minimize the risks of errors when compared to stainless steel instruments.^2,7,8,12,17^ At the same time, NiTi rotaries are prone to fracture.^16,18^ Presently some studies have assessed the ability of NiTi rotary endodontics in pulpectomy of deciduous teeth,^7-10,19-24^ However, different approaches have been tested in research to evaluate canal instrumentation in permanent teeth.^6,11-17,25-29^

In 2008, a new approach with only one NiTi instrument based on the reciprocating motion was proposed by Yared.^11,13,16,30,31^ This technique has increased in popularity with the marketing of newly designed reciprocating NiTi instruments, including Reciproc and WaveOne.^16,25,30^ These instruments are made of M-wire NiTi alloy and mounted on a dedicated handpiece and motor to operate the reciprocating rotation.^14,26^ The counterclockwise rotation cuts dentin and the reversing clockwise movement releases the file from the canal wall.^25^ Moreover, the characteristic design of blades permits the operator to apply low instrumentation force coupled with low risk of iatrogenic errors.^16,18,30^

Reciprocating systems seem to be applicable for preparation of curved canals in primary molars. Furthermore, the use of reciprocating motion was shown to decrease preparation time, simplifying the procedure, promoting patient cooperation with similar shaping ability in comparison with continuous rotation.^18^ All of these are desirable properties and particularly important considerations in pediatric patients.

Recently, the efficacy of rotary files in cleaning, shaping, time of preparation and instrument distortion in root canal therapy of primary teeth has exhibited greater potential versus manual files.^7^ Moreover, Soares et al^9^ reported that in primary teeth, rotary instrumentation provided superior canal cleanliness, requiring less time for completion of canal preparation in comparison to manual instrumentation. On the other hand, the results of a study by Madan et al^10^ conducted on primary teeth showed significantly efficient cleaning of only coronal thirds by using ProFile versus K-file. In the above-mentioned study, ProFile prepared canals in significantly longer time compared to K-file. Franco et al^13^ observed that in permanent teeth, the application of NiTi files is more time-consuming by using reciprocating movements in comparison to continuous rotary motion. However, in another study by Paqué et al^11^ the shaping ability of reciprocating and rotary approaches were similar, with faster reaching of the working length (WL) with reciprocating motion.

Currently, no data are available on the performance of reciprocating systems in narrow and curved root canals of primary teeth. Given the importance of validation of a novel single file armamentarium and its research interest, the aim of the current study was to assess root canal cleaning and shaping efficacy and the time taken by manual, rotary instrumentation, and engine-driven files under reciprocating movement for canal preparation in primary molars.

## Methods

The protocol of this study was approved by Research Review Board and Ethics Committee of Zahedan University of Medical Sciences (code: 6628).

For collecting specimens used in this study, all the freshly extracted primary mandibular secondary molars were cleaned, washed and immersed in 1% chloramine-T solution until all the samples were selected according to inclusion criteria. The inclusion criteria included: 1) no previous treatment, with fully formed apices; 2) 20-40° curvature of mesiobuccal canal; 3) no signs of calcification, internal and external root resorption in the mesiobuccal canal; 4) type III canals in the mesial root according to Weine category.^32^ Items 2-4 were confirmed with preoperative anatomic images obtained by cone-beam computed tomography (CBCT).

From the collected teeth, a total of 64 primary mandibular second molars were selected. These selected teeth were then kept in distilled water until further use. This sample size (16 teeth in each group) was driven based on power of 0.9 and α error of 0.05 by using a previously published study. The mesiobuccal canal was used for the study.

The teeth were washed under tap water. Thereafter, a standard access cavity was created by using high-speed diamond-coated burs (Dentsply, Maillefer, Ballaigues, Switzerland) to establish straight-line access for file insertion. All the specimens were then irrigated with 5 mL of 1.0% sodium hypochlorite delivered in a syringe with a 27-gauge needle (Endo Eze; Ultradent Products Inc, South Jordan, UT). By inserting a #10 K-file (Dentsply, Maillefer, Ballaigues, Switzerland), root canal patency was confirmed. Subsequently, the working length (WL) was recorded preoperatively by subtracting 1 mm from the length of the #10 K-file when tip visualized at the apical foramen. Subsequently, the root canals were washed with normal saline, dried and colored with India ink dye applied until it leaked from the foramen. The ink dye was re-injected into the canals to assure complete dye penetration throughout the canals. Furthermore, a #10 K-file was again inserted into the canals to assure no bubble formation. Then the selected teeth (roots and apices) were mounted in the cold-cured acrylic resin blocks to perform canal preparation in unbiased circumstances and stored under wet condition at room temperature.

Afterwards, the teeth were serially numbered and randomly divided into four groups (three experimental and one control), including 16 samples per group by applying table of random numbers. Reciproc, available on the market for use in reciprocating rotation, Mtwo, one continuous rotary file, and stainless steel K-file were evaluated in the current study. Group I was treated using K-files, group II using Mtwo and group III using Reciproc. Group IV was used as a control.

**Group I:** Canal preparations were performed with K-files (Dentsply, Maillefer, Ballaigues, Switzerland). The canal was prepared to #25 MAF (Master Apical File) followed by stepping back to #40.

**Group II:** Mtwo files (VDW GmbH, Munich, Germany) were used for canal preparation. The adopted file sequence was 10/0.04, 15/0.05, 20/0.06 and 25/0.06. The instruments were used in 250 rpm continuous rotation with constant low torque setting (1.5 N.cm) to the full length of the canal.

**Group III:** Instrumentation was performed with a single Reciproc 25/0.08 (VDW GmbH, Munich, Germany) in reciprocating movement.

In groups II and III, the files were applied in light pecking motion during instrumentation until they reached full WL. All the engine-driven instruments were activated by a VDW.SILVER RECIPROC, Sirona Endo Motor (VDW GmbH, Munich, Germany). Before the experiment started, microscopic inspection (Pico, Zeiss, Oberkochen, Germany) for defects or deformities was performed for each instrument. In each experimental group, the instruments were covered with RC-Prep (Premier Dental Products, USA) and then used for canal instrumentation. All the files were discarded after only four canal instrumentation procedures. In all the experimental groups, irrigation with 1 mL of 1.0% sodium hypochlorite (Clorox Professional Products, Oakland, CA) and then 1 mL of normal saline was carried out after each instrument change. A final copious flush with 10 mL of saline was accomplished after canal preparation was completed. Canal patency was verified following the use of each file with #10 K-file.

**Group IV:** No instrumentation was carried out in this group.

One single operator experienced in NiTi instrumentation technique performed all the stages of the research in the Department of Pediatric Dentistry of Zahedan Dental School. Only five canals were treated every time to prevent the operator fatigue. Then, the teeth were retrieved from the acrylic resin blocks. The pulp chambers were filled and the root apices were covered with sticky wax. Subsequently, the samples were evaluated by using light microscopy and postoperative CBCT analysis.

### Cleaning efficacy 

For evaluation of cleaning efficacy, decalcification, dehydration and clearing processes were carried out. First, the specimens were placed separately in daily-renewed fresh 7% chloric acid until they were completely decalcified. The samples were then washed under running water. To dehydrate the teeth, the samples were immersed in 70% and then 80% ethyl alcohol. The immersion time was 16 h for each and the solutions were renewed every 8 h. The immersion was followed by 90% and 100% alcohol each for 3 h with hourly renewed solution. Then, the specimens were cleared while immersed in methyl salicylate (Merck, Germany) for 6 h. Finally, ink removal was carefully examined under a stereomicroscope (Nikone SMZ800, Japan) at ×10 in the cervical, middle and apical thirds and scored from 0 to III as follows:

Score 0: thorough ink removal

Score I: nearly thorough ink removal (remains of ink detected)

Score II: partial ink removal (india ink remained in some areas)

Score III: no ink removal (a considerable amount of ink remained).

Evaluation of shaping ability and cleaning efficacy were performed by a blinded examiner with no prior knowledge of each experimental group to avoid any bias.

### Shaping ability

Gradual decrease in the canal width from the coronal to the apical third of the canal was counted as good taper, whereas, other than that described above was recorded as poor taper.

### Time of preparation 

The time taken for preparation of each root canal (including time needed for instrumentation, instrument change and irrigation) was measured by a digital chronometer to the nearest 0.1% seconds.

### Instrument failure 

After every use, the files were examined under microscopic magnification for deformation or fracture. If file deformation or fracture occurred, a new instrument was substituted.

### Statistical analysis 

Analysis of data was carried by using SPSS 19.0 for Windows (SPSS Inc., Chicago, Illinois, USA). Data of shaping ability were analyzed using chi-squared and Fisher’s exact tests. Because of the ordinal nature of cleaning efficacy, the data of this variable were statistically analyzed with nonparametric methods. Since normality assumptions of preparation time were not verified, Kruskal-Wallis test was used to investigate data of preparation time for inter-group comparisons. Post hoc tests were used to determine which group was statistically different from the others. Statistical significance was set at P < 0.05.

## Results

The control group demonstrated a cleaning efficacy score of III in all the thirds. In addition, this group did not exhibit any good taper. For data analysis of cleaning efficacy in the experimental groups, each third was considered separately. In all the thirds, considering the mean rank of cleaning efficacy, the best result was achieved with Reciproc, in which a lower mean rank demonstrated good efficacy and vice versa. However, statistical analysis demonstrated no significant difference in the apical third cleaning efficacy (P = 0.749). In the middle third, cleaning efficacy of Reciproc was significantly better than that of K-file (P = 0.005). In statistical analysis, no significant difference was detected between Reciproc and Mtwo (P = 0.477), and between Mtwo and K-file (P = 0.191). However, in the coronal third, despite the same performance of Reciproc and Mtwo files (P = 0.080), both yielded better results than manual instrumentation (P < 0.05).

As illustrated in Table 1, the number of samples with postoperative good taper was greater for Mtwo, followed by Reciproc and K-file in descending order. In relation to shaping ability, statistical analysis showed significant differences between the groups (P = 0.006). The frequency of good canal taper was significantly higher either in continuous rotary or reciprocating groups compared with the hand file group (P < 0.05). In relation to canal taper, the results yielded by Mtwo were slightly better than those yielded by Reciproc, whereas no significant difference was observed between these engine-driven groups (P = 1.00).

**Table 1:** Number (%) of cases in different cleaning efficacy and shaping ability criteria, and mean (SD) values for preparation time in K-file, Mtwo and Reciproc groups

**Groups**	**Cleaning efficacy** **n (%)**												**Shaping ability** **n (%)**		**Preparation time (seconds)**
	**Apical Third**				**Middle Third**				**Coronal Third**				**Good taper**	**Poor taper**	
	**0**	**I**	**II**	**III**	**0**	**I**	**II**	**III**	**0**	**I**	**II**	**III**			
**K-files**	2 (12.5)	6 (37.5)	7 (43.7)	1 (6.25)	0 (0.0)	5 (31.2)	10 (62.5)	1 (6.25)	1 (6.25)	14 (87.5)	1 (6.25)	0 (0.0)	5 (31.2)	11 (68.8)	179.85 (66.27)
**Mtwo**	2 (12.5)	8 (50.0)	4 (25.0)	2 (12.5)	3 (18.7)	5 (31.2)	8 (50.0)	0 (0.0)	9 (56.2)	7 (43.7)	0 (0.0)	0 (0.0)	13 (81.2)	3 (18.8)	53.75 (12.57)
**Reciproc**	1 (6.25)	10 (62.5)	5 (31.2)	0 (0.0)	4 (25.0)	8 (50.0)	4 (25.0)	0 (0.0)	11 (68.7)	5 (31.2)	0 (0.0)	0 (0.0)	12 (75.0)	4 (25.0)	17.34 (10.41)
**P value**	0.749				0.022				0.001				0.006		<0.001

## Discussion

Currently, engine-driven instrumentation is of great interest with promising findings. In numerous studies, good canal tapering, sufficient apical enlargement and less procedural errors of NiTi instruments have been endorsed.^7,17,24^ NiTi instruments, as clearly known, have some disadvantages, including high cost of the handpiece and files. Moreover, the operator needs to be experienced clinically. Regarding the performance of engine-driven systems in primary teeth, some data have been published in which different systems other than Mtwo and Reciproc have been compared.^7-10,19-24^ In primary teeth, canal cleaning and shaping is mandatory to achieve successful treatment results,^16^ as required in permanent teeth. Therefore, we evaluated the efficacy of continuous rotary movements using Mtwo and reciprocating rotary movement with the application of Reciproc for root canal preparation of primary molars. Since manual K-file has been traditionally used for canal preparation of deciduous teeth, the authors decided to apply the traditional preparation as a reference for comparison.

The analysis for time necessary for preparation is also illustrated in Table 1. The average time taken to prepare each canal was the shortest for Reciproc, with K-file group being the longest. Significant differences were revealed in preparation time between the groups (P < 0.001). The results of post-test revealed differences between each two groups (P < 0.05). No instrument deformation or fracture was observed throughout the study.

Various approaches have been applied to assessing cleaning efficacy of different preparation systems. In our study, we used ink injection and clearing technique for this purpose. Including the control group also confirmed that the irrigation solution could not remove dye solely.

Overall, Mtwo and Reciproc, particularly the latter, resulted in good cleanliness. However, Reciproc created a cleaning efficacy statistically equal to that by Mtwo at all the thirds. Data analysis of coronal region revealed significantly lower efficacy of K-file than others, whereas in the apical third, no significant differences were found. However, Reciproc showed the highest frequency of grades 0 and I and the lowest of grades II and III in the apical third. In the middle third, analysis revealed a significant difference between Reciproc and K-file. Katge et al^24^ in accordance with our findings reported no significant difference in the apical third using WaveOne and ProTaper. In the two other thirds, in their study, WaveOne showed better cleaning efficacy.

The characteristics of files such as cross-section and cutting flutes should be considered as main factors for cleaning as well as tapering efficacy. We decided to use Mtwo based on a previous study^8^ in which Mtwo was reported as being efficient for primary molar canal preparation. In addition, good canal curvature preservation together with low preparation events have been attributed to Mtwo.^12^

In reciprocating movement, Reciproc cuts in both directions, clockwise and counterclockwise, resulting in a balanced force. In the present study, reciprocating system with only one file, though it is contrary to instrumentation protocol used routinely, made the canal sufficiently clean, followed by Mtwo with a non-significant difference. Reciproc yielded significantly superior performance while assessing in the coronal and middle regions than K-file. Mtwo also created efficient root canal preparation, especially in the coronal third, as it was emphasized in one former study.^8^

In line with our results, several authors^7,9,10,24^ have not reported superiority of K-file as compared with continuous and reciprocating rotary. Some studies, in contrast to ours, demonstrated that manual and NiTi files clean the canal with the same efficiency.^8,12,27^ However, we revealed the better performance of Mtwo in the coronal third. But as a whole and despite the similarity of our results to others, we emphasize that there were various methodological differences between the current and previous studies, including the techniques applied to evaluate canal cleaning, the type of rotary system, the sequence and number of instruments used etc.

According to some previous studies, no complete apical third cleaning may be achieved with hand or engine-driven filing.^8,10,24,33^ In addition, as disclosed in the present study, cleaning was not completely performed in the apical third. By using stereomicroscopic assessment, apical thirds of groups presented no differences in cleanliness. There is worldwide agreement regarding the fundamental effect of the apical third cleaning on the success rate of endodontic procedures in permanent teeth.^28^ In other words, there is often a challenge to clean the apical area. But, due to several accessory canals in the coronal and middle portions in primary dentition, the cleaning of apical third is not challenging as it is in permanent teeth.

The results of cleaning efficacy may also be explained by intracanal anatomic configuration.^28^ Although the primary molars uniformly exhibited buccolingually widened canals,^3^ it might be inferred that toward the coronal region the canal cross-section might favor the action of continuous rotary and reciprocating systems. The increasing diameter of dentinal tube and the subsequent dentin softness toward the coronal third,^28^ when faced with rotary instrumentation, either in continuous or reciprocating motion, may be perceived as another contributing factor. In addition, the presence of apical ramification helps us interpret the insignificant difference in the apical third.

In some reports, making impressions of canal space has been considered as a tool for analysis of the canal taper,^7,22^ The authors evaluated canal shaping ability of different instruments by CBCT.

In this research, there was a slightly higher frequency of good canal taper with Mtwo compared with Reciproc. However, both groups resulted in a more tapering shape in comparison to K-file. The results are also in line with Musale and Mujawar,^7^ Crespo et al,^20^ and Nagaratna et al,^22^ who compared rotary files with K-files. The answer to why the tapering obtained by K-file is poorer than engine-driven instrumentation achieved either with Reciproc or with Mtwo is the number of continuous or reciprocating motions per unit of time that NiTi instruments make to shape the canal. Additionally, cutting efficacy of blades might be postulated as another factor. The S-shaped cross-section of Mtwo and Reciproc^8,16^ increase engagement of the cutting side of file to primary tooth dentin, possibly enhancing tapering toward apex.

Although the analysis of canal taper and cleaning efficacy showed no differences between Mtwo and Reciproc, the analysis of preparation time obviously demonstrated that these two groups differed from each other. Mtwo was more time-consuming compared with Reciproc. Furthermore, the results showed that K-file was the slowest and did not indeed save time clinically, which is one prerequisite for keeping the child cooperative throughout the procedure. In addition, reduced time of preparation would decrease practitioner fatigue.

The time necessary for root canal preparation is the subject of many investigations.^7,8,10,13,19-23^ Although the root morphology of primary and permanent teeth differ significantly, the findings with permanent teeth can be cited for comparison. The time recorded in our study was shorter than the mean value reported for permanent teeth in the literature,^11-13,22^ perhaps due to less dentin density,^12,24^ shorter root canal and fewer files required. In several studies the time needed with rotary and reciprocating files was shorter than hand files,^12^ consistent with our results.

Another study, in line with ours, indicated shorter preparation time with Mtwo compared with K-files.^12^ The factor contributing to shorter preparation time of Mtwo might be superior cutting efficiency.^12^ In the present study, as expected, and also in studies of Katge et al^24^ and Paqué et al,^11^ the preparation time of single-file technique was the lowest for Reciproc obviously because of no need to change instruments. Most studies reported that rotary instrumentation was significantly faster than manual filing.^7,8,12,20,22^ Conversely, in one study the preparation time for K-file was shorter than the rotary, which was explained by operator’s skill.^10^

It should be emphasized that faster instrumentation does not necessarily lead to good outcomes. In pediatric dentistry, the variability of preparation time is correlated to patient’s cooperation and fatigue on one side and quality, success and prognosis of treatment on the other side. Due to the dual effect of reducing the time, we considered variables of shaping and cleaning efficacy as more important factors determining the quality of preparation.

The number of times we used each instrument was four based on studies reviewed.^8,12,6^ Probably, it is the primary reason for no evidence of file deformation or fracture in the present study. The periodically reversing motion decreases torsional and flexural stress, subsequently decreasing taper lock and fracture.^24,30^ For NiTi instruments, the reduced tendency to screw in and the torque-limited handpiece have been also proposed as the cause for low rate of fracture.

In vitro instrumentation, due to its great difference from clinical circumstances is one limitation and one potential confounder that should be taken into account in future research. It is also of great importance to emphasize that the results derived from our study cannot be generalized to other instrumentation files with different characteristic designs.

Attempts were made to ensure matching of the groups. Since variations in canal curvature before preparation may affect the outcome of different instrumentations performed in this study, we restricted samples to those with canal curvatures of 20‒40°. Also we distributed them randomly into different groups. Hence group balancing regarding preoperative canal curvature was assumed. In order to eliminate the confounding effect of canal type, we also used the mesiobuccal canal only. Performing the study on real canals of human teeth allowed us to experience clinical situation as far as possible because of the similarity in canal cross-section, the dentin hardness and its surface texture to clinical condition.

Likewise, the teeth were mounted with exposed crowns to match the holding condition of samples during instrumentation as well as simple handling. However, since preparations were performed with different file designs, care was exercised to ensure that nearly identical preparation was used in the study groups. Therefore, we made sure that the final apical size of the samples was nearly equivalent to #25 file. One experienced practitioner carried out the procedures for the purpose of standardization.

In spite of the aforementioned disadvantages for NiTi instruments, the results achieved in primary teeth as emphasized in our study, are rewarding and encouraging. According to the design used in the present study and considering preparation time, reciprocating instrumentations using Reciproc and continuous rotary movements using Mtwo are good suggestions for canal preparation in deciduous teeth. Amongst them, Reciproc is the most suitable file for canal preparation of primary teeth. However, further investigations, especially clinical ones, are required to confirm clinical outcomes.

## Conclusion

Based on the results of the present study, we concluded that good canal taper was significantly reported in favor of Reciproc and Mtwo than K-file. By using rotary movements, especially reciprocating movements, good cleaning efficacy was achieved. There was also significant reduction in time of preparation with Reciproc and Mtwo than with K-file. In comparison of Reciproc and Mtwo, significantly more time was saved with Reciproc.

## Acknowledgements

This article was based on a research protocol approved by the Deputy of Research and Technology of Zahedan University of Medical Sciences. Accordingly, the authors would like to thank the Deputy of Research and Technology for their financial support.

## Authors’ contributions

NR, MR, AM and FA defined the intellectual content and designated the research protocol. Sample preparation and data acquisition was performed by FE. NR also analyzed and interpreted the data and drafted the manuscript. The whole manuscript was critically reviewed and edited by all the authors for important intellectual content. All authors have read and approved the final manuscript.

## Funding

Zahedan University of Medical Sciences funded the research.

## Competing interests

The authors declare that they have no competing interests with regards to authorship and/or publications of this paper.

## Ethics approval

Ethics Committee of Zahedan University of Medical Sciences approved the study protocol (code: 6628).
